# Interaction of aquaporin 4 and N-methyl-D-aspartate NMDA receptor 1 in traumatic brain injury of rats

**DOI:** 10.22038/IJBMS.2018.29135.7037

**Published:** 2018-11

**Authors:** Li-Hua Chen, Hong-Tian Zhang, Ru-Xiang Xu, Wen-De Li, Hao Zhao, Yi Yang, Kai Sun

**Affiliations:** 1 The Affiliated Bayi Brain Hospital, The PLA Army General Hospital, Beijing 100700, China

**Keywords:** Aquaporin 4, Edema, N-methyl-D-aspartate NMDA receptor, NMDAR1, Traumatic brain injury

## Abstract

**Objective(s):**

methyl-D-aspartate NMDA receptor (NMDAR) and aquaporin 4 (AQP4) are involved in the molecular cascade of edema after traumatic brain injury (TBI) and are potential targets of studies in pharmacology and medicine. However, their association and interactions are still unknown.

**Materials and Methods:**

We established a rat TBI model in this study. The cellular distribution patterns of AQP4 after inhibition of NMDAR were determined by Western blotting and immunoreactive staining. Furthermore, the regulation of NMDA receptor 1 by AQP4 was studied by injection of a viral vector targeting AQP4 by RNAi into the rat brain before TBI.

**Results:**

The results suggest that AQP4 protein expression increased significantly (*P*<0.05) after TBI and was down-regulated by the NMDAR inhibitor MK801. This decrease could be partly reversed using the NMDAR agonist NMDA. This indicated that AQP4 mRNA levels and protein expression are regulated by the NMDA signaling pathway. By injection of AQP4 RNAi viral vector into the brain of TBI rat models, we found that the mRNA and protein levels of NMDAR decreased significantly (*P*<0.05). This suggested that NMDAR is also regulated by AQP4.

**Conclusion:**

These data suggested that the inhibition of AQP4 down-regulates NMDAR expression, which might be one of the mechanisms involved in edema after TBI.

## Introduction

Considering the incidence, prognosis, and expenses of treatment of traumatic brain injury (TBI), efficient neuroprotective therapies are necessary. Results of approximately 30 randomized controlled clinical trials were unsatisfactory (1, 2), largely owing to the complex pathogenesis of TBI. It is difficult to find a new therapy for TBI without understanding the details of the cellular and molecular post-traumatic processes of TBI pathophysiology (3).

The N-methyl-D-aspartate NMDA receptor (NMDAR) has been considered very important in the pathological sequence of traumatic brain injury (4). Numerous experimental studies have shown the beneficial effects of NMDA receptor antagonist in central nervous system trauma and/or ischemia. However, it is still not clear whether the activation or suppression of NMDAR after TBI is beneficial for the alleviation of edema (4-6). Of the 6 kinds of the aquaporins expressed in the brain, aquaporin 4 (AQP4) is the most widely studied and is involved in potential mechanisms for cerebral edema (7). AQP4 facilitates the flow of water from blood capillaries into the brain parenchyma and then into astrocytes in cytotoxic edema, it is also the channel for the outflow of water in vasogenic edema (8). AQP4-null mice are protected from cellular (cytotoxic) brain edema produced by water intoxication, brain ischemia, or meningitis (9).

Although NMDAR and AQP4 are important factors for the occurrence and development of brain traumatic edema, their interactions are poorly understood. There is some evidence that AQP4 might be regulated by excessive excitatory amino acid (EAA) (10). Following traumatic cortical contusions in rats, the levels of AQP4 mRNA were significantly higher at the site of injury than those at remote sites of the same brain (11, 12). Also, the levels of AQP4 expression correlate with the degree of brain edema as seen by magnetic resonance imaging (MRI) (13). Gunnarson et. al. have recently shown that the activation of group I metabotropic glutamate receptors (mGluRs), which are endogenous to astrocytes, increased AQP4 permeability for water (14). Based on this indirect evidence demonstrating that AQP4 and NMDAR1 are inherently associated, we developed a hypothesis that these two important factors in TBI regulate each other.

In the present study, we have examined the link between AQP4 and NMDAR1 after TBI. First, we established the rat TBI model. Then, the regulation and distribution patterns of AQP4 in the TBI model after inhibition of NMDAR were examined by Western blotting and immunoreactive staining. Furthermore, the regulation of NMDA receptor 1 by AQP4 was studied using RNAi via a viral vector targeting AQP4 in the rat brain before TBI. The mRNA and protein expression levels of NMDAR1 were examined by immunohistochemistry and quantitative real-time polymerase chain reaction (real time-PCR).

**Figure1:**
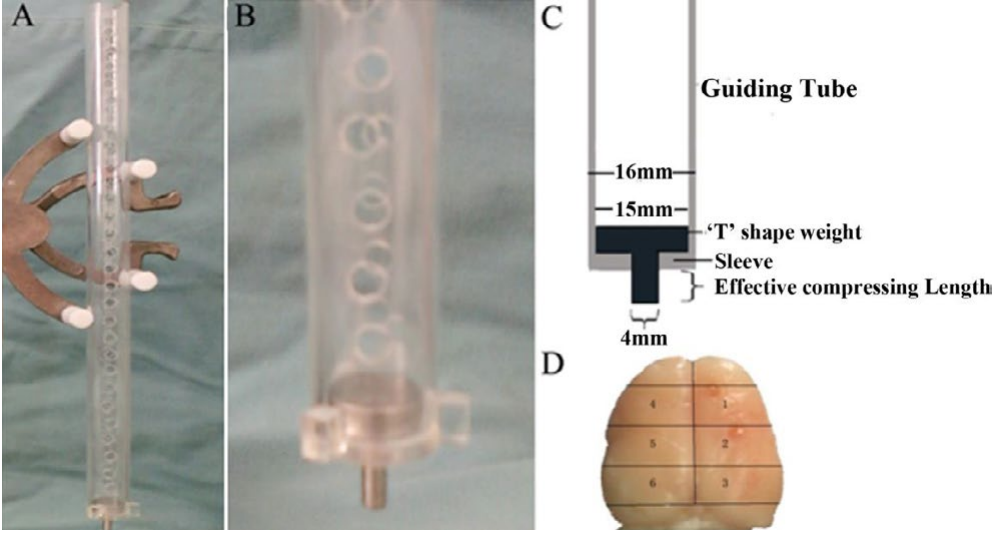
The injury animal model. A: The device for injury model; B: The contusion was made by free falling of 20 grams “T” shaped weight at 30 cm height; C: The guiding tube is 30 cm long. Its inner diameter is 15 mm and the outer diameter is 16 mm. A maximum of 2 or 4 mm depression of the brain surface was allowed; D: The demarcation of the brain was made by dividing the brain into ipsilateral or contralateral to the injury. The right side is the ipsilateral side of injury and was divided into three areas, pre-impact (area 1), impact (area 2), and post-impact (area 3). The injury contra-lateral side was divided into three areas also, the contra-pre-impact (area 4), contra- impact (area 5), and contra-post-impact (area 6)

## Materials and Methods

***Animals and groups***

Male Sprague-Dawley rats, weighing between 260 and 280 grams were used in our study. All animals were anesthetized with 3.5% halothane in 70% N_2_ and 30% O_2_ and maintained at 0.3% halothane using a facemask. In the experiment testing the edema produced by 2 mm or 4 mm footplates, and the effect of MK801 on the development of edema, fifteen rats were used and divided into 3 groups, the sham group received craniotomy only; the control group received craniotomy and the injury; the MK801 group were injured and given MK801. This study was carried out in strict accordance with the recommendations in the Guide for the Care and Use of Laboratory Animals of the National Institutes of Health. The animal use protocol has been reviewed and approved by the Institutional Animal Care and Use Committee (IACUC) of Chinese PLA Army General Hospital.

In the experiment testing the regulation of expression of AQP4 by MK-801 and NMDA, fifty rats were divided into 5 groups. The sham group (receiving only craniotomy); the control group (receiving craniotomy and injury); the MK801 group (injured and administered MK801); the NMDA group (injured and administered NMDA); and the MK801+NMDA group (injured and administered MK801+NMDA). In each group, 5 rats were perfused for immunohistochemical staining, and the other 5 rats were decapitated and their brains were used for Western blotting analysis.

In the experiments testing the regulation of NMDAR1 by AQP4, using RNA interference targeting AQP4, twenty male rats were used for the 4 RNAi viruses targeting rat AQP4. The viral vectors targeted different DNA sequences of AQP4. Viral vectors were injected into the brains of 5 animals.

***Establishment of TBI rat model***

The TBI model in this study used a modified form of the weight-drop technique (15). The contusing device consisted of a 40 cm long (4 mm diameter) guiding tube made of polyethylene plastic (Figures 1A, B). The wall of the tube was perforated with 5 mm diameter airholes at 1 cm intervals to prevent the influence of air compression (ref) (Figure 1B). The device was kept perpendicular to the surface of the skull and guided a falling weight onto the surface of the dura. “T” shaped footplate, which can be stopped by the sleeve of the guiding tube that is designed to avoid variable mechanical puncture of the dura (16, 17). After falling, the inferior surface of “T” shaped footplate was in direct contact with the exposed tissue. A “T” shaped footplate was used to produce more stable TBI in rats. Both the footplates with 2 mm and 4 mm compress lengths, led to neuronal damage in the cortex ipsilateral to the impact. The footplate with 4 mm compress length damaged the entire cortex and the shorter footplate only damaged the upper layers of the cortex.

Each rat was placed in a stereotactic frame and craniotomy of the right hemisphere was performed. The diameter of the bone flap removed was 4.5 mm, and the center of the skull hole caused by trepanation was positioned at 1.5 mm posterior and 2.5 mm lateral to the bregma. A standardized parietal contusion was made by allowing a 20-g weight to fall onto the footplate from a height of 30 cm (9). The compression towards the brain tissue of the “T” shaped footplate was determined by its effective compressing length (Figure 1C).

We used two footplates with the effective compressing lengths of 2 mm and 4 mm. A heating lamp was used to avoid hypothermia during the surgery. The heart rate and respiration of the animals were also monitored during the surgery. The hemisphere including contusion was divided into 3 parts; interior to impact (area 1), impact (area 2), and posterior to impact (area 3). Area 2 was the epicenter of TBI injury. The other hemisphere was divided into 3 parts; contralateral interior to impact (area 4), contralateral impact (area 5), and contralateral posterior to impact (area 6). The thickness of all brain sections was 4 mm (Figure 1D). After impact, the bone flap was replaced and sealed with bone wax, the scalp was then sutured and the animals were allowed to recover. Twenty-four hours after the injury, tissue samples were obtained from the brain for analysis.

***BBB permeability***

Rats were anesthetized and the left femoral vein was identified under the operation microscope, and 3 ml/kg of 2% Evans blue solution was administered intravenously. Twenty-four hours after the infusion, the rats were perfused and the brains were removed. Fresh tissue samples collected from the impact area were weighed and then homogenized with 50% trichloroacetic acid (TCA). After centrifugation at 15000 rpm for 20 min, the absorbance of the samples was measured at 615 nm.

***Water content measurement***

Six regional brain samples were obtained from each rat. All samples were quickly removed and processed rapidly for measurement of water content. Samples were heated at 110 ^°^C for 24 hr. Water content was calculated using the formula: H_2_O (%)=(wet weight-dry weight)/ wet weight (%).

**Table 1:** The real-time polymerase chain reaction primers

Name	Sequence (5’-3')	Length
AQP4 (F)	CGGAGCCAGCATGAATCC	100 bp
AQP4 (R)	AGCGCCTATGATTGGTCCAA	
NMDAR1 (F)	GCGCGGACCATGAATTTT	100 bp
NMDAR1 (R)	CGTTCCACTCCTTTTTGTTGCT	
Rat β-actin (F)	GGAGATTACTGCCCTGGCTCCTA	150 bp
Rat β-actin (R)	GACTCATCGTACTCCTGCTTGCTG	

***Regulation of AQP4 by MK801***

To determine whether N-methyl-D-aspartate (NMDA) antagonists mitigate primary damage to the brain induced by head trauma in rats, dizocilpine (MK-801, Sigma), a noncompetitive NMDA antagonist, and NMDA (Sigma), an agonist, were used. MK801 was administered IP with a dose of 30 mg/kg, 30 min before and 20 min after TBI. NMDA was administered 30 min after TBI. In the MK801+NMDA group, the first dose of MK801 was administered 30 min before TBI. And the second dose of MK801 and NMDA were administered 30 min after the injury.

***Regulation of NMDAR1 by AQP4 RNAi***

**Figure 2:**
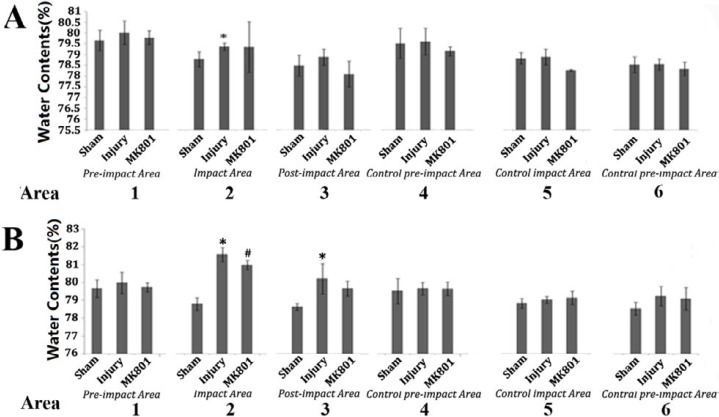
The water contents in different groups. A: Traumatic brain edema by the footplate with 2 mm effective compress length; B: Traumatic brain edema of the brain injured by the footplate with 4 mm effective compress length

Sequence information for AQP4 RNAi and corresponding nonspecific controls used in this study is provided in Table 1. We prepared 4 RNAi plasmids containing different RNAi sequences (Figures 2A, B). The packaging of the virus was done by Genechem 

Corporation (www.genechem.com.cn). The viral vectors were named LV1, LV2, LV3, and LV4. The titer of all of the viruses was tested in 293T cells and found to be as high as 10^9 ^TU/µl.

After validation of RNAi effect *in vivo*, we found that three of the four viral vectors were effective in reducing both the mRNA and protein levels of AQP4 *in vivo*. Brain tissues transfected for RNAi were selected for the NMDAR1 assay. NMDAR1 gene transcription and protein expression after RNAi and samples from animals injected with non-specific viral vectors were analyzed by real-time PCR and Western blotting. To validate RNAi caused by lentiviruses *in vivo*, we injected the viruses into the striatum of rats at the site of impact. Animals were immobilized on a stereotaxic apparatus. A scalp incision was made; using a hand-held drill, a hole (1 mm in diameter) was made in the skull and the dura mater was opened using the bent end of a hypodermic needle. A Hamilton syringe containing the viral preparation was inserted using anteroposterior and lateral coordinates assigned to the CA1 region of the hippocampus. We used the following coordinates: anterior-posterior, 4 mm; lateral, 2.5 mm, vertical: 2.4 mm, for injection in the CA1 pyramidal neuron layer of the hippocampus. The lentiviral preparation was injected using a Harvard Apparatus injection pump at a flow rate of 0.1 μl/min to minimize tissue damage. After injecting the lentiviral preparation, the cannula was removed slowly at a rate of 0.5 mm/min and the skin was sealed with biological glue. Animals were then returned to their cage and allowed to recover. A heating lamp was used to avoid post-operative hypothermia.

**Table 2:** Target sequence information for aquaporin 4 RNAi virus

Target sequence of APQ4	Sequence, sense (5-3')	Start position
No.1	GCGTGGGATCCACCATTAA	221
No.2	GCATTGCCACCATGGTTCA	305
No.3	CCACGGTTCATGGAAACCT	509
No.4	GCTGTGATTCCAAACGGAC	221

Thirty minutes after the administration of AQP4 RNAi lentivirus, animals were subjected to traumatic brain injury, and 24 hr later, the animals were sacrificed and the brain tissues of the impact area were analyzed. We prepared 4 RNAi viral vectors. Each lentivirus targeted different DNA sequences of AQP4. To validate the effect of RNA interference, AQP4 mRNA levels and protein expression of tissues from the animals injected with AQP4 RNAi lentivirus or non-specific RNAi lentivirus were analyzed by real-time PCR and Western blotting.

***Real-time PCR***

According to the manufacturer’s instructions using the RNeasy Micro kit (Qiagen, Hilden, Germany), 50 ng of total RNA was reverse-transcribed (1 hr at 37 ^°^C) into first-strand cDNA using oligo-dT primers in a reaction volume of 20 μl with Sensiscript Reverse Transcriptase (Qiagen).An aliquot of cDNA (1 µl) was used in PCR containing 0.2 µm both forward and reverse primers, 1× PCR buffer, 1.5 mm MgCl_2_, 0.1 mm dNTP mix, and Taq DNA polymerase (Qiagen). The cDNA was amplified using 30 PCR cycles with an initializing step of 3 min at 95 ^°^C, DNA denaturation at 95 ^°^C for 30 sec, annealing at 55 ^°^C for 30 sec, and elongation at 72 ^°^C for 1 min. RT-PCR samples were then separated electrophoretically on a 1.5% agarose gel containing ethidium bromide and visualized under UV-light. The real-time PCR primers are summarized in Table 2.

**Figure 3:**
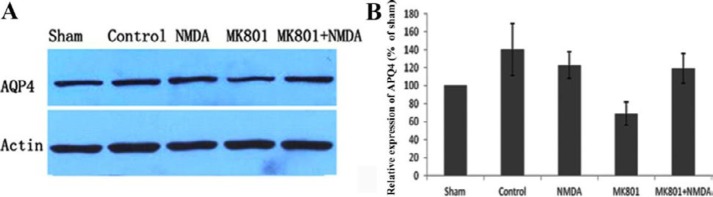
The protein expression of aquaporin 4 in different groups. The representative immunoblotting demonstrating AQP4 expression levels of the impact area in the 5 groups, sham, control, MK80, NMDA, and MK80+NMDA, at 24 hr time points after injury; B: Analysis of AQP4 protein expression. The intensity level for each band relative to GAPDH was determined, and the value of the sham group was assigned as 100%. Compared with the sham group, the control group showed a significant increase in AQP4 expression (140±28%, *P*<0.05). With the treatment of MK801 before and immediately after the injury, the AQP4 protein expression was strongly down-regulated to 68±12%. The effect of MK801 could be reverted to 119±16% by the administration of NMDA after MK801. Furthermore, we didn’t find significant changes in AQP4 expression in the injured area in the NMDA group, which received NMDA before the injury when compared with the sham group

**Figure 4:**
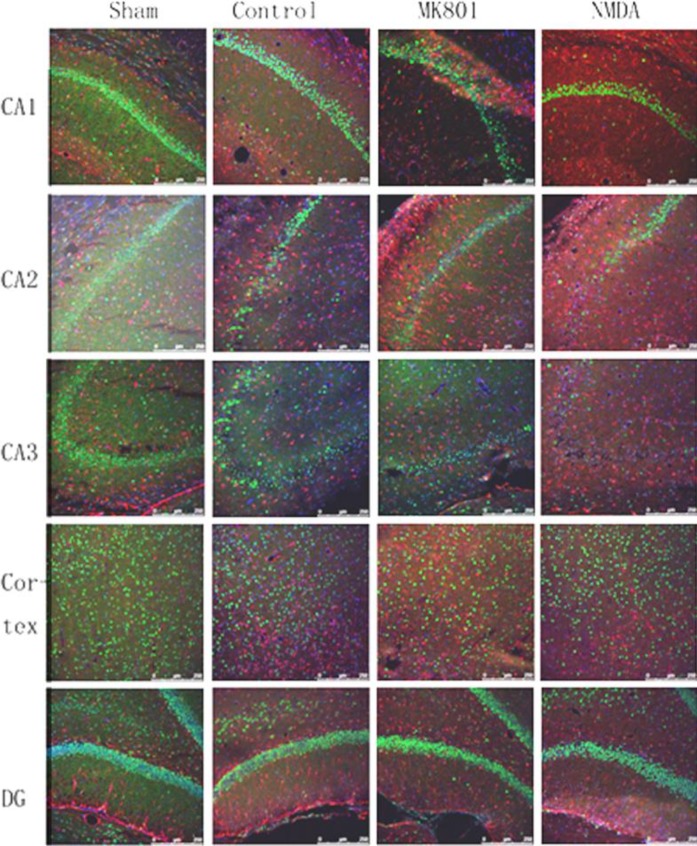
Representative immunofluorescent staining of aquaporin 4 in the hippocampus at the impact area of the brain (scale bars, 250 μm)

Quantification of gene transcripts was performed with the Opticon II system (MJ Research, MA, USA) using the SYBR Green I real-time PCR kit (Takara, Japan). All expression values were normalized against GAPDH. All reactions were performed in duplicates, with at least three technical and three biological replicates.

***Western blotting***

 Brain tissue was homogenized on ice in radioimmunoprecipitation (RIPA) buffer containing proteolysis inhibitors and protease inhibitors. Homogenates were centrifuged at 13,500 ×g, 4 ^°^C for 30 min to remove nuclei and to harvest supernatants. The protein concentration of each supernatant was determined using a protein assay kit (Bio-Rad, Hercules, CA, USA). Samples were adjusted to the same concentrations and 15 µg of each sample was loaded onto 10% Bis-Tris polyacrylamide gels for electrophoresis. The protein was then transferred to a nitrocellulose membrane (Invitrogen, Carlsbad, CA). After that, membranes were blocked at 25 ^°^C for 45 min in Tris-buffered saline plus Tween-20 (TBS-T) containing 3% milk and then incubated overnight at 4 ^°^C in the same buffer with a mouse anti AQP4 monoclonal antibody (Abcam, Inc. Cambridge, MA) diluted 1:1000. The membrane was washed and then incubated for 2 hr with a horseradish peroxidase (HRP)-conjugated goat anti-mouse IgG, diluted 1:5000 in TBS-T plus 3% milk. Enhanced chemiluminescence (ECL) system (Amersham, Buckinghamshire, UK) was used for detection of bands, and densitometric analysis was used to quantify AQP4 protein expression levels by determining intensity values for each band relative to GAPDH.

***Immunohistochemistry***

Sections of area 2 were processed for AQP4 and NeuN immunohistochemistry. Followed by three washes of 5 min each in phosphate-buffered saline (PBS), pH 7.4, sections were blocked in a solution containing PBS and 1% goat serum for 45 min in a humid chamber. Sections were further incubated with mouse anti-NeuN (1:400, Chemicon) and rabbit anti AQP4 (1:400, Chemicon) in PBS containing 1% goat serum and 0.25% Triton X-100 for 24 hr. After washing, sections were incubated for 2 hr with cy2-conjugated goat anti-mouse IgG (ImmunoJackson; 1:200), and Texas Red-conjugated goat anti-rabbit IgG (ImmunoJackson; 1:200), followed by a 15 min incubation with DAPI (Sigma; 1:1000). The fluorescence was visualized using a standard confocal microscope.

***Pixel intensity and staining density analysis***

Using pre-established gain and offset settings that ensured that all pixels within any given section fell within the photomultiplier detection range (no desaturated or oversaturated pixels in any tissue section), at least 6 low magnification images of each area, CA1, CA2, CA3, frontal cortex, and dentate gyrus of hippocampus, were obtained using the confocal microscope with a 10× objective, images were collected from all sections without altering confocal settings. In each image, pixels positive above background were subsequently selected and recorded, then the averaged pixel intensity for all positive pixels was calculated. The relative intensity was indicated by the average pixel intensity for pixels above background and normalized to that of the sham group.

***Statistical analysis***

Statistical analysis of the results was performed using analysis of variance (K-W nonparametric ANOVA) followed by post-ANOVA (S-N-K) test to compare differences among individual groups. Data with *P*<0.05 were considered statistically significant.

## Results

***Brain permeability***

No changes in the counts of pyramidal cells were detected in hippocampal subfield CA1 in the ipsilateral hemisphere of impact. Contralateral cortex and hippocampus remained unaffected.

The blood-brain barrier (BBB) permeability, determined by means of Evans blue concentration in the tissue (μg dye/gram wet tissue), was 15.143±4.329 (µg/ gram sample) with a 2 mm footplate, and 24.199±5.597 (µg/gram sample) with a 4 mm footplate. The BBB leakage was significantly increased at the impact area of the injured hemisphere with the longer hitting footplate (*P*<0.05).

**Figure 5:**
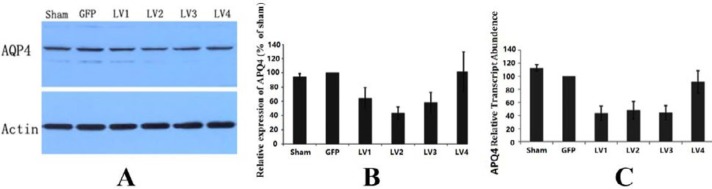
Validation of the RNA interference virus of aquaporin 4. A: The decrease of AQP4 in protein expression was observed *in vivo* after the administration of AQP4 targeting RNAi virus, numbered as LV1, LV2, and LV3. The sequence in LV4 did not significantly lower AQP4 expression; B: Intensity values for each band relative to GAPDH were evaluated by semi-quantify AQP4 protein expression levels. Protein expression level of AQP4 in the control group which was infected by the virus containing the same backbone of RNAi virus but not AQP4 targeting sequence was assigned as “100%”. The four RNAi lentiviruses, LV1, LV2, LV3, and LV4, lowered the expression of AQP4 to 64.42±14.83%, 43.22±8.91%, 58.33±14.21%, and 102.13±27.57% of the control group. Except for LV4, the other 3 viruses all lowered AQP 4 significantly (*P*<0.05, n=5); C: The mRNA transcription of AQP4 *in vivo* after RNAi by lentiviruses LV1, LV2 and LV3 and LV4 were also decreased. The transcript level of AQP4 in the control group which was infected by the virus containing the same backbone of RNAi virus but not AQP4 targeting sequence was assigned as “100%”. Compared to the control group, the lentiviruses LV1, LV2, and LV3 significantly decreased the transcription of AQP4 to 43.29±11.10%, 48.02±13.11%, and 44.26±10.84% (*P*<0.05, n=5). The decreasing trend of LV4 on AQP4 transcription (91.11±17.46%) did not reach significance

***Water content***

As shown in Figure 2A, edema caused by the 2 mm footplate was 79.36±0.16% in area 2, the impact area, which was significantly higher than that of the sham group, which was 78.78±0.35%. Although the water content of all the other brain areas was higher than that of the sham group, the difference was not statistically significant. Furthermore, the water content of the impact area after the treatment with MK801 was 79.75±1.17%, which was not significantly different as compared with the TBI control group.

The footplate with a 4-mm effective impact length resulted in severe edema of the brain (Figure 2B). The water contents of areas 2 and 3 were 78.35±0.64% and 78.75±0.41%, respectively, and were both significantly higher than the water contents of the sham group. As compared to the control group, the cerebral water content was reduced both at the area of impact (78.02±2.17%) and at the area posterior to the impact (79.81±2.54%) of the injured hemisphere after treatment with MK801, and this reduction was statistically significant only in the impact area (*P*<0.05).

The water content at the area of impact (77.91±2.27%) and at the area posterior to impact (83.15±3.34%) of the injured cerebral hemisphere of the NMDA treatment group did not show any significant difference as compared to that of the control group.

The water content of the contralateral hemisphere of the damage was not influenced by TBI with either 2 mm or 4 mm footplates in the TBI model.

**Figure 6:**
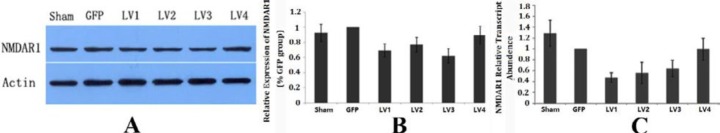
The protein expression of N-methyl-D-aspartate NMDA receptor 1 in different groups. A: The protein expression and transcript abundance of NMDAR1 in the impact area of the rat brain after AQP4 RNA interference *in vivo*. The protein expression of NMDAR1 was reduced significantly by LV1, LV2, and LV3 RNAi virus injection (*P*<0.05, n=5); B: Relative intensity values for each band were analyzed. Protein expression level of NMDAR1 in the control group, which was infected with the virus containing the same backbone of RNAi virus but not AQP4 targeting sequence was assigned as “100%”. The three RNAi lentiviruses, LV1, LV2, and LV3, lowered the expression of AQP4 significantly in the control group (*P*<0.05, n=5); C: The mRNA transcript of NMDAR1 also decreased in the tissues treated by AQP4 knockdown induced by LV1, LV2, and LV3 (*P*<0.05, n=5)

***Regulation of AQP4 by MK801***

Edema in the impact area was attenuated by MK801, thus, only the tissue from the impact area was selected for Western blotting and immunohistochemistry for AQP4. AQP4 protein expression of the TBI control group in the impact area significantly increased up to 140.27±28.96% as compared to that of the sham, which was assigned as 100%. After the administration of MK801, AQP4 protein expression was reduced significantly to 68.97±12.75% at 24 hr after injury. Reduction in AQP4 expression due to treatment with MK801 was recovered partly to 119.18±16.25% by treating the animals with NMDA 20 min after MK801 administration (Figures 3A, B). AQP4 protein expression was not altered 24 hr after injury upon administration of NMDA 30 min before the injury. We observed positively stained cells for AQP4 in the areas CA1, CA2, CA3, cortex, and the dentate gyrus. Immunostaining data for which pixel intensity was applied in AQP4 fluorescence measurements, AQP4 staining of CA1, CA2, and CA3 in the TBI control group was relatively higher than those of the sham group, indicating an increase in the expression of AQP4. Morphology of the positively stained cells indicated that AQP4 was mainly localized in the astrocytes (Figure 4). AQP4 staining could be recovered significantly as compared to that of the MK801 group upon treating the animals with NMDA after MK801 administration.In the cortex and the dentate gyrus, TBI control group did not show any significant difference in AQP4 staining as compared with that of the sham group. Treatment with MK801 or MK801 and NMDA did not significantly alter average levels of AQP4 protein determined by fluorescence staining 24 hr after brain trauma.

***Regulation of NMDAR1 by AQP4 RNAi***

 Three of the four RNAi sequences targeting AQP4 inhibited AQP4 gene transcription* in vivo* by at least 70% relative to the control, as determined by real time-PCR analysis.

To confirm the down-regulation of AQP4 protein at the expression at the, we performed Western blot analysis on normal rat brain injected with AQP4 RNAi lentivirus or the corresponding control virus. Twenty-four hours after injection, AQP4 protein expression was markedly reduced in samples from animals that were transfected with the AQP4 RNAi lentivirus. However, protein expression showed a slight change in samples from animals that were transfected with a non-specific control virus (Figures 5A-C).

Two of the most suitable sequences targeting non-overlapping sites of AQP4 were used in experiments in order to down-regulate AQP4 expression *in vivo*. To confirm the effect of RNAi, we used two independent sequences that produced similar levels of mRNA knockdown (at least 70%) in rat brains. Both RNAi constructs reduced AQP4 transcription and protein expression, which led to a corresponding decrease in NMDAR1 transcriptional activity and protein expression.

 NMDAR1 transcription was inhibited 24 hr after transfection with RNAi constructs targeting AQP4 DNA sequences (Figures 6A-C). Protein expression of NMDAR1 was reduced significantly by injection of LV1, LV2, and LV3 RNAi viral vectors (*P*<0.05, n=5). Relative intensity values for each band were analyzed. Protein expression levels of NMDAR1 were assigned as 100% for the control group injected with the viral vector comprising the identical backbone as that of the RNAi lentivirus, except for the AQP4 targeting sequence. The three RNAi lentiviruses, LV1, LV2, and LV3, significantly reduced expression of AQP4 in the control group (*P*<0.05, n=5). mRNA transcription of NMDAR1 was decreased in tissues treated by AQP4 knockdown induced by LV1, LV2, and LV3 (*P*<0.05, n=5).

## Discussion

In the present study, the rat TBI model was established and these rats were subjected to different treatments in groups. Distribution patterns of AQP4 after inhibition of NMDAR were examined. Furthermore, the regulation of NMDA receptor 1 by AQP4 was studied by injection of an RNAi lentivirus targeting AQP4 in the rat brain before TBI. The association of AQP4 and NMDA receptor 1 is described in previous sections. The study revealed several findings.

We first found that the regulation of AQP4 after brain injury in the area of impact is closely related to NMDAR. This is because brain damage results in excessive extracellular concentrations of the excitatory neurotransmitter, glutamate, which also elicits a significant increase in expression of AQP4 24 hr after brain trauma in the area of impact; Moreover, we found that the water content increased significantly at the area of impact 24 hr after the impact. Administration of MK801, only in the area of impact, revealed that edema was reduced. When rats were administered NMDA shortly after the injury, the increase of AQP4 expression after the injury was notably reduced as compared to that in animals that received the injury only. Signaling through NMDAR is important for up-regulation of AQP4 in the impact area, as indicated by a significant decrease in AQP4 expression by inhibition of NMDAR. However, NMDA was not the only factor which contributed to the increase in AQP4 expression because the administration of NMDA alone did not stimulate up-regulation of AQP4. NMDA induced up-regulation of AQP4 only along with some other factors released because of brain injury. AQP4 functions as a water-selective channel in the plasma membranes of cells and is the common route for the entry and exit of water, and inhibition of AQP4 hampers the efflux of water during brain edema. AQP4 plays an important role in the development of “cytotoxic” models of brain edema in cerebral ischemia, hyponatremia, and meningitis. Therefore, selective inhibition of AQP4 may help in reducing cytotoxic edema in these clinical conditions (6). AQP4 is also important for the removal of water in vasogenic edema, caused by brain tumor and brain abscess. In such cases, AQP4 knockdown or inhibition aggravates brain edema (9, 18). It was traditionally considered that, because of the opening of BBB, brain edema after TBI is mainly vasogenic in and around the impact area. However, accumulating experimental data using TBI models has revealed that traumatic brain edema is mainly cytotoxic (19). We found that MK801 reduces brain edema, by down-regulating protein expression and gene transcription of AQP4. In most of the brain trauma models, brain edema develops 24–48 hr after injury (20), and cytotoxic edema is prevalently observed in such cases. Furthermore, AQP4 commonly functions as the channel for water flow into the cells during edema, and inhibition or down-regulation of AQP4 is helpful in attenuating brain edema. Taken together, the protective effect of MK801 on our brain trauma injury model is at least partly owing to the down-regulation of AQP4.

We have found that the responses of AQP4 in CA1, CA2, CA3, cortex, and dentate gyrus of the impact area are not the same after impact and after administration of MK801 or NMDA. Similar to the tissue from the impact area, the AQP4 expression of CA1, CA2, and CA3 increased significantly after the injury and reduced with MK801 treatment after the injury. This increase was attenuated significantly upon NMDA administration to the animals before the trauma. However, the expression pattern of AQP4 in the cortex and the dentate gyrus was completely altered. One of the reasons for this may be the damage to the cortex. The integrity of the cortical tissue and BBB was damaged because of the impact and could not be rescued during the 24 hr following injury. Another reason for the change in the expression pattern of AQP4 may be the non-uniform distribution of NMDA receptors, both in numbers and types. It is well recognized that the subfield CA3 contains relatively less NMDA than AMPA/KA receptors (21-23).

This study also revealed that the down-regulation of AQP4 by RNAi after TBI also decreased the expression of NMDAR1. The decrease in AQP4 protein expression in approximately 60% of the control group after RNAi was not as significant as that in the AQP4 transcription, which was reduced to 40–50% of the GFP control group. This may partly be owed to the fact that we used brain tissues as samples for our experiments. The cells transfected by the RNAi virus are only a part of entire tissues. Brain injury can induce dynamic changes in NMDAR subunit expression and reiterate the functional consequences of NMDAR subunit alterations (24). The underlying mechanism includes calcium signaling. Protein kinases modulate the activity of several ligand-gated ion channels, including the NMDA subtypes of glutamate receptors (25). This regulation is achieved through Ca^2+^-dependent phosphatase. Furthermore, Thrane *et al*. demonstrated that brain swelling induced by hypoosmotic stress triggers Ca^2+^ signaling in astrocytes and that, the deletion of the AQP4 gene markedly interferes with these events [25]. This suggested that AQP4 not only serves as an influx route for water but also is critical for initiating downstream signaling events, such as Ca^2+^ signaling.

Although the mechanism underlying the regulation of NMDAR1 by AQP4 is still unknown, these data support that AQP4 could be considered a putative target for the treatment of TBI in pharmacology, as the inhibition of AQP4 down-regulates NMDAR1, which is one of the major components of the damage signaling pathway after TBI.

## Conclusion

These data suggest that the inhibition of AQP4 down-regulates NMDAR, which may be one of the mechanisms involved in edema after TBI.

## Conflicts of Interest

The authors declare that they have no conﬂicts of interest.

## References

[1] Tolias CM, Bullock MR (2004). Critical appraisal of neuroprotection trials in head injury: what have we learned?. NeuroRx.

[2] Schouten JW (2007). Neuroprotection in traumatic brain injury: a complex struggle against the biology of nature. Curr Opin Crit Care.

[3] Quillinan N, Herson PS, Traystman RJ (2016). Neuropathophysiology of brain injury. Anesthesiol Clin.

[4] Carvajal FJ, Mattison HA, Cerpa W (2016). Role of NMDA receptor-rediated glutamatergic signaling in chronic and acute europathologies. Neural Plast.

[5] McIntosh TK, Vink R, Soares H, Hayes R, Simon R (1990). Effect of noncompetitive blockade of N-methyl-D-aspartate receptors on the neurochemical sequelae of experimental brain injury. J Neurochem.

[6] Biegon A, Fry PA, Paden CM, Alexandrovich A, Tsenter J, Shohami E (2004). Dynamic changes in N-methyl-D-aspartate receptors after closed head injury in mice: Implications for treatment of neurological and cognitive deficits. Proc Natl Acad Sci U S A.

[7] Badaut J, Lasbennes F, Magistretti PJ, Regli L (2002). Aquaporins in brain: distribution, physiology, and pathophysiology. J Cereb Blood Flow Metab.

[8] Xu M, Su W, Xu QP (2010). Aquaporin-4 and traumatic brain edema. Chin J Traumatol.

[9] Papadopoulos MC, Verkman AS (2007). Aquaporin-4 and brain edema. Pediatr Nephrol.

[10] Faden AI, Demediuk P, Panter SS, Vink R (1989). The role of excitatory amino acids and NMDA receptors in traumatic brain injury. Science.

[11] Verkman AS (2001). Applications of aquaporin inhibitors. Drug News Perspect.

[12] Vizuete ML, Venero JL, Vargas C, Ilundáin AA, Echevarría M, Machado A (1999). Differential upregulation of aquaporin-4 mRNA expression in reactive astrocytes after brain injury: potential role in brain edema. Neurobiol Dis.

[13] Zhang C, Chen J, Lu H (2015). Expression of aquaporin-4 and pathological characteristics of brain injury in a rat model of traumatic brain injury. Mol Med Rep.

[14] Gunnarson E, Zelenina M, Axehult G, Song Y, Bondar A, Krieger P (2008). Identification of a molecular target for glutamate regulation of astrocyte water permeability. Glia.

[15] Clark RS, Schiding JK, Kaczorowski SL, Marion DW, Kochanek PM (1994). Neutrophil accumulation after traumatic brain injury in rats: comparison of weight drop and controlled cortical impact models. J Neurotrauma.

[16] Chen JQ, Zhang CC, Jiang SN, Lu H, Wang W (2016). Effects of Aquaporin 4 knockdown on brain edema of the uninjured side after traumatic brain injury in rats. Med Sci Monit.

[17] Shapira Y, Shohami E, Sidi A, Soffer D, Freeman S, Cotev S (1988). Experimental closed head injury in rats: mechanical, pathophysiologic, and neurologic properties. Crit Care Med.

[18] Zhang M, Cui Z, Cui H, Cao Y, Zhong C, Wang Y (2016). Astaxanthin alleviates cerebral edema by modulating NKCC1 and AQP4 expression after traumaticbrain injury in mice. BMC Neurosci.

[19] Unterberg AW, Stover J, Kress B, Kiening KL (2004). Edema and brain trauma. Neuroscience.

[20] Bareyre F, Wahl F, McIntosh TK, Stutzmann JM (1997). Time course of cerebral edema after traumatic brain injury in rats: effects of riluzole and mannitol. J Neurotrauma.

[21] Blixt J, Svensson M, Gunnarson E, Wanecek M (2015). Aquaporins and blood-brain barrier permeability in early edema development after traumatic brain injury. Brain Res.

[22] Bernert H, Turski L (1996). Traumatic brain damage prevented by the non-N-methyl-D-aspartate antagonist 2,3-dihydroxy-6-nitro-7-sulfamoylbenzo[f] quinoxaline. Proc Natl Acad Sci U S A.

[23] Goebel DJ, Poosch MS (1999). NMDA receptor subunit gene expression in the rat brain: a quantitative analysis of endogenous mRNA levels of NR1Com, NR2A, NR2B, NR2C, NR2D and NR3A. Brain Res Mol Brain Res.

[24] Chen JH, Yang LK, Chen L, Wang YH, Wu Y, Jiang BJ (2016). Atorvastatin ameliorates early brain injury after subarachnoid hemorrhage via inhibition of AQP4 expression in rabbits. Int J Mol Med.

[25] Lieberman DN, Mody I (1994). Regulation of NMDA channel function by endogenous Ca(2+)-dependent phosphatase. Nature.

